# Neoadjuvant Radiochemotherapy Alters the Immune and Metabolic Microenvironment in Oral Cancer—Analyses of CD68, CD163, TGF-β1, GLUT-1 and HIF-1α Expressions

**DOI:** 10.3390/cells13050397

**Published:** 2024-02-25

**Authors:** Manuel Weber, Jutta Ries, Kristina Braun, Falk Wehrhan, Luitpold Distel, Carol Geppert, Rainer Lutz, Marco Kesting, Leah Trumet

**Affiliations:** 1 Department of Oral and Cranio-Maxillofacial Surgery, Uniklinikum Erlangen, Friedrich-Alexander-Universität Erlangen-Nürnberg (FAU), Glückstraße 11, 91054 Erlangen, Germanyleah.trumet@uk-erlangen.de (L.T.); 2 Deutsches Zentrum Immuntherapie (DZI), Comprehensive Cancer Center Erlangen-EMN (CCC ER-EMN), Friedrich-Alexander-Universität Erlangen-Nürnberg (FAU), 91054 Erlangen, Germany; 3 Private Office for Maxillofacial Surgery, 09599 Freiberg, Germany; 4 Department of Radiation Oncology, Friedrich-Alexander-Universität Erlangen-Nürnberg (FAU), 91054 Erlangen, Germany; 5 Institute of Pathology, Friedrich-Alexander-Universität Erlangen-Nürnberg (FAU), 91054 Erlangen, Germany; carol.geppert@uk-erlangen.de; 6 Department of Operative Dentistry and Periodontology, Friedrich-Alexander Universität Erlangen-Nürnberg (FAU), 91054 Erlangen, Germany

**Keywords:** macrophage polarization, immune tolerance, hypoxia, metabolism, induction therapy, RCT, OSCC, oral squamous cell carcinoma, HNSCC, head and neck squamous cell carcinoma

## Abstract

Background: The first-line treatment of oral squamous cell carcinoma (OSCC) involves surgical tumor resection, followed by adjuvant radio(chemo)therapy (R(C)T) in advanced cases. Neoadjuvant radio- and/or chemotherapy has failed to show improved survival in OSCC. Recently, neoadjuvant immunotherapy has shown promising therapeutic efficacy in phase 2 trials. In this context, the addition of radio- and chemotherapy is being reconsidered. Therefore, a better understanding of the tumor-biologic effects of neoadjuvant RCT would be beneficial. The current study was conducted on a retrospective cohort of patients who received neoadjuvant RCT for the treatment of oral cancer. The aim of the study was to evaluate the influence of neoadjuvant RCT on the immunological tumor microenvironment (TME) and hypoxic and glucose metabolisms. Methods: A cohort of 45 OSSC tissue samples from patients were analyzed before and after RCT (total 50.4 Gy; 1.8 Gy 5× weekly; Cisplatin + 5-Fluorouracil). Immunohistochemistry for CD68, CD163, TGF-β, GLUT-1 and HIF-1α was performed using tissue microarrays and automated cell counting. Differences in expression before and after RCT and associations with histomorphological parameters (T-status, N-status) were assessed using the Mann–Whitney U test. Results: Tumor resection specimens after neoadjuvant RCT showed a significant decrease in CD68 infiltration and a significant increase in CD163 cell density. The CD68/CD163 ratio was significantly lower after RCT, indicating a shift toward M2 polarization. The GLUT-1 and HIF-1α expressions were significantly lower after RCT. Larger tumors (T3/T4) showed a lower GLUT-1 expression. Other biomarkers were not associated with the T- and N-status. Conclusions: Neoadjuvant RCT with 50.4 Gy induced a shift toward the M2 polarization of macrophages in the TME. This change in immune composition is not favorable and may be prognostically negative and counteract immunotherapeutic approaches. In addition, the decreased expressions in GLUT-1 and HIF-1α indicate reductions in the glucose metabolism and hypoxic energy metabolism in response to “high dose” neoadjuvant RCT, which may be therapeutically desirable.

## 1. Introduction

The introduction of immune checkpoint inhibitors (ICIs) has redefined the role of immunotherapy (IT) in the treatment of solid malignancies, making it the fourth pillar of cancer treatment alongside surgery, radiotherapy (RT) and chemotherapy [1]. Despite its outstanding successes in a small number of patients, it is becoming increasingly clear that the majority of cancer patients do not respond adequately to ICI treatment alone [2]. Therefore, combining ICI treatment with another immune-activating agent is a promising approach for IT in solid malignancies [2]. In recent years, there has been a paradigm shift in the understanding of the mechanism of action of RT. In the past, RT was seen as a local therapy aimed at inducing tumor cell death while sparing the surrounding tissue. Today, the immunomodulatory properties of RT are increasingly becoming the focus of scientific interest [3]. It is now believed that RT can act as an “in situ vaccination” against the patient’s tumor [3,4,5].

The 5-year survival rate of head and neck squamous cell carcinoma (HNSCC), including oral squamous cell carcinoma (OSCC), has not been significantly improved over the past 30 years despite the introduction of multimodal treatment approaches [2]. The standard of care for OSCC is primary surgical tumor resection with the concomitant removal of the regional lymph nodes. Histological parameters (pTNM, depth of infiltration) are used to decide on adjuvant RT/radiochemotherapy (RCT), which is required in a relevant proportion of patients [6]. The routine RT or RCT of HNSCC consists of a dose of 60 Gy or 70 Gy applied in a normofractionated manner with 2 Gy per day, five times per week, resulting in a total treatment duration of 6 or 7 weeks [5]. The current German treatment guideline for OSCC recommends these doses for the definitive radiotherapy and a reduced dose of 54 and 66 Gy in the adjuvant setting [6]. This may be combined with chemotherapy in high-risk cases [6].

The importance of immunological parameters for the prognosis of OSCC has been demonstrated in our previous work [7,8,9,10,11]. The design of early trials of neoadjuvant radiotherapy in OSCC did not take into account the immunomodulatory effect of RT. Total doses between 50 Gy and 20 Gy were applied in the studies by Mohr et al., Kessler et al. and Mücke et al [12,13,14]. These studies show a slightly improved survival in patients treated with neoadjuvant RCT compared to the adjuvant-treated control group and acceptable toxicity. Due to methodological weaknesses and thus the lack of formal evidence, these studies on neoadjuvant RCT were not included as treatment recommendations in the current German treatment guideline for OSCC [6]. The study by Kessler et al. compared neoadjuvant RCT with adjuvant RT alone in OSCC [12]. The allocation to the two groups was not randomized. Instead, those patients who could not receive neoadjuvant RCT due to renal or cardiac conditions were assigned to adjuvant RT as the control group. The results of the study show a significantly better survival in the neoadjuvant cohort [12]. However, due to the study design, the survival benefit cannot be attributed to the neoadjuvant approach. Thus, the addition of chemotherapy or simply the better initial condition of the patients in the RCT group could be responsible for the improved survival. Although the study by Kessler et al. could not prove the superiority of neoadjuvant treatment in OSCC, the samples obtained in this study can be used to analyze the biological effect of neoadjuvant RCT in OSCC. A previous study on this patient cohort could show slight decreases in CD8 and Granzyme B positive effector T-cells with a significant reduction in FoxP3 positive regulatory T-cells, indicating a shift toward effector T-cells [15]. These data support an immune-activating effect of neoadjuvant RCT.

However, in addition to activating the immune system, RT also has immunosuppressive effects [3,16]. This is mediated, among other mechanisms, by inducing the expression of the immunosuppressive cytokine transforming growth factor beta (TGF-β) and the immune checkpoint ligand programmed cell death 1 (PD-L1). TGF-β-mediated immunosuppressive effects are clinically relevant at high doses of ionizing radiation [4,17], which are routinely used in the adjuvant setting as well as in the early neoadjuvant studies of Kessler, Mohr and Mücke [12,13,14]. TGF-β is a cytokine associated with the M2 polarization of macrophages [18]. M2 polarized, tumor-promoting macrophages express TGF-β and the cytokine can also drive macrophage polarization toward M2 [18]. In our previous work, we showed that M2 macrophages are strongly associated with the development and progression of OSCC [8,9,11,19]. CD68 is a surface marker for all macrophages, including anti-tumoral M1-polarized macrophages, whereas M2 cells can be identified using the CD163 antigen [20,21].

In addition to immunological factors, there are several metabolic parameters that are relevant to tumor progression. A hypoxic microenvironment contributes to radio resistance in cancer [22]. Hypoxia-inducible factor 1 alpha (HIF-1α) is a major marker of hypoxic conditions [22]. Through its role as a transcription factor, HIF-1α can induce the gene expression of several hypoxia-associated genes, including glucose transporter 1 (GLUT-1) [23]. In OSCC, a high HIF-1α expression is associated with glucose metabolism, increased aggressiveness and tumor progression [24]. The Warburg Effect describes the dependence of cancer cells on glucose metabolism, which is also observed in OSCC [25]. GLUT-1 leads to increased glucose uptake and the up-regulation of glycolysis [22]. The glucose carrier GLUT-1 was found to be overexpressed in OSCC tissue, and its levels correlated with tumor stage, poor prognosis and resistance to therapy [25]. Preclinical studies indicate an increased response of OSCC cells to chemotherapy and RT if the GLUT-1 expression is reduced [25]. In addition, cells with a high GLUT-1 expression showed increased resistance to cisplatin [25].

There is only little information available on the immune-modulatory and metabolic effects of neoadjuvant RCT [26]. This is particularly true for OSCC. Therefore, it was the aim of the current study to analyze the effect of a 50.4 Gy normofractionated RCT with 5-FU and cisplatin on macrophage polarization (CD68 and CD163), the TGF-β expression and the hypoxic and glucose metabolism (GLUT-1 and HIF-1α) in OSCC tissue.

## 2. Materials and Methods

### 2.1. Patient Collective

For this study, tissue samples from 45 OSCC patients that received neoadjuvant radiochemotherapy (RCT) prior to surgical tumor resection and were aged between 38 and 71 years at the time of sampling were analyzed. All patients were treated at University Hospital Erlangen. Tissue sampling included biopsies prior to RCT and tumor resection specimens after RCT. All patients were classified according to their TNM status, grading and staging before and after neoadjuvant RCT [12]. Detailed information on the number of cases in each subgroup as well as clinical and histomorphological parameters can be found in Table 1.

All patients with a year of diagnosis between 1997 and 2003 were included. The use of formalin-fixed paraffin-embedded material from the archive of the Institute of Pathology was approved by the Ethics Committee of the Friedrich–Alexander University of Erlangen–Nuremberg on 24 January 2005 (21_ 19 B), waiving the need for consent for using existing archived material.

### 2.2. Therapy

The therapeutic approach for OSCC in this cohort differed from the current standardized therapeutic protocol. The patient cohort analyzed in the current analysis was treated as previously described [12]: neoadjuvant RCT followed by surgery was applied [12,15]. After the diagnosis of OSCC, a pan endoscopy was performed, and during this procedure, the tumor-bearing area was tattooed at a safety margin of 15 mm around the visible or palpable tumor area [12]. During neoadjuvant RCT, two chemotherapy cycles were applied on days 1–5 and 29–33 with 5-FU as a 120 h intravenous infusion (800 mg/m^2^/d) and cisplatin as a short intravenous infusion (20 mg/m^2^/d) prior to radiotherapy. External radiation therapy was performed on the primary tumor as well as on the regional lymph nodes with 6 MV photon radiation. Ionizing radiation was conventionally fractionated by 1.8 Gy 5 times weekly, accumulating to a total dose of 50.4 Gy. The target volume was the primary tumor site as well as the lymphatic drainage in the ipsilateral neck. In total, 4–6 weeks after neoadjuvant RCT, the surgical tumor resection, simultaneous microvascular reconstruction and neck dissection (ND) were performed. The ND was carried out as a modified radical neck dissection with the removal of lymph node levels I–V, as described by Robbins et al., on the tumor-affected side as well as a suprahyoid ND including levels I–III on the contralateral side [12].

### 2.3. Sampling

For the analysis of the tissue samples, a tissue microarray (TMA) from a previous project of the Department of Radiation Oncology and the Department of Oral and Maxillofacial Surgery, both University Hospital Erlangen, Friedrich–Alexander Universität Erlangen–Nürnberg, Erlangen, Germany, was used [15]. The samples were grouped into biopsy and tumor resection specimens. TMA samples, with a 2 mm wide core size per sample, were generated manually using a TMA-Grandmaster (3D-Histech, Budapest, Hungary). A TMA block had a maximum size of 6 × 10 tissue spots. Then, 2 µm sections were taken from each TMA using a rotary microtome (HistoCore AUTOCUT, Leica Biosystems, Nussloch, Germany). Afterward, they were fixed in a heat cabinet at 57 °C on glass slides (Superfrost Plus Gold Adhesion Microscope Slides, White Tab, Epredia, Portsmouth, NH, USA) and subsequently examined histopathologically. The slides were thereafter prepared for immunohistochemical (IHC) staining, as described in the following.

First, the 2 µm TMA slides were deparaffinized. Subsequently, the slides were rehydrated through a descending alcohol series. For quality reasons, after that procedure, the slides were not placed in an unsubmerged state at any timepoint to avoid dry-out and the consequent risk of a high background density due to nonspecific antibody binding. For epitope unmasking, antibody-specific heat-induced epitope retrieval procedures were performed according to the manufacturer’s protocol. For each staining process, a specific antigen retrieval procedure had to be performed. For Glut1 (ab0084; dilution, 1:400) and CD163 (NCL-L; dilution, 1:3000; concentration, 3.1 g/L), samples were heated at 99 °C in citrate buffer (pH 6.0) for 30 min, followed by cooling down at room temperature for 30 min and 5 min in DAB wash puffer (DAB (DAB-Kit 957D-30 Medac)). The staining protocol of HIF-1α (ab51608; dilution, 1:2000; concentration, 0.961 mg/mL), TGF-β1 (ab215715; dilution, 1:250; concentration, 0.568 mg/mL) and CD68 (DAKO M0814; dilution, 1:3000; concentration, 185 mg/L), the heat-induced epitope retrieval procedure was performed using EDTA buffer (pH 9.0) at 99 °C for at least 20 min followed by cooling down for the same time at room temperature and 5 min in DAB wash puffer (DAB (DAB-Kit 957D-30 Medac). After that procedure, the immunohistochemical staining of the aforementioned markers was conducted according to the manufacturer’s protocol using an Auto Stainer Plus (Dako cytomation, Aligent, Santa Clara, CA, USA) also using the DAB (DAB-Kit 957D-30 Medac) staining kit. Finally, all histological slides were covered with Aquatex (Merck 1.08652.0050).

Human tissues, the exact type depending on the staining protocol, such as tonsil, spleen and lymph node tissue, were stained in each run as positive controls. Human mucosa, stained only with antibody diluent, served as the negative control.

After the aforementioned procedure, followed by the digital scanning (Panoramic 250 Flash III, 3D Histech, Budapest, Hungary) of the TMA-slides, each slide was analyzed using QuPath 0.2.3 [27].

**Figure 1:**
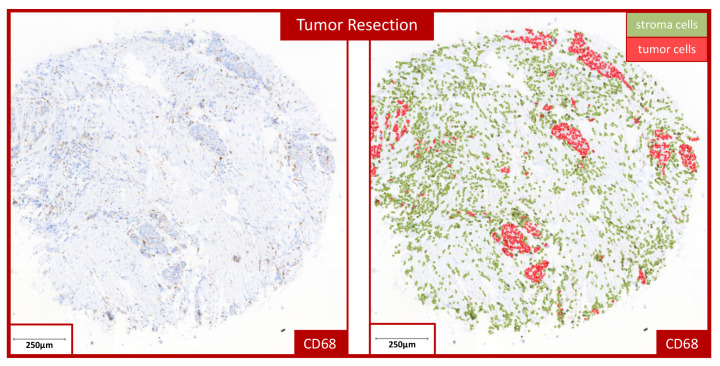
Example of automated cell counting in a tumor resection specimen. The image shows an example of the automated cell counting used to determine the labeling index (LI) in a tumor resection specimen stained for CD68. The left micrograph shows the stained TMA sample. The right micrograph displays a visualization of the cell counting performed in the QuPath software (https://qupath.github.io/). All detected cells allocated to the stroma compartment are marked in green, and cells allocated to the epithelial tumor compartment are indicated in red.

For automated cell counting, a TMA grid was created in QuPath and adapted to the individual samples. Simple tissue detection was then performed to distinguish between stroma and epithelium. This was followed by “positive cell detection”, which calculated the number of positive cells present—meaning those expressing the marker. By training the “object classifier”, the most accurate possible analyses could be performed, which could be verified with the help of pathological specialists. The “object classifier” makes it possible to assign the detected cells to different cell types. This makes it possible to differentiate between the tumor-free stroma and epithelial parts of the tumor tissue and to evaluate the respective values separately (Figure 1). The “train object classifier” tool was used to manually mark the different tissue compartments for the training of the software.

The labeling index (LI) could then be calculated from the values obtained. The LI was determined using QuPath, which calculated the percentage of positive cells from all counted cells. The LI was determined in stroma, epithelium and overall (Figure 1), where overall means we added the epithelial and stromal parts of each TMA sample.

When evaluating and assessing the samples, it should be noted that after therapy, samples were classified as tumor resection; it was often no longer possible to detect epithelium. Therefore, we analyzed all three compartments to avoid interpretation mistakes. In our study, we concentrated the analysis on the stroma and overall compartments.

### 2.4. Statistical Analysis

First, we performed an exploratory data analysis. As there was no normal distribution in our cohort, we performed the nonparametric Mann–Whitney U test to determine whether expression levels differed (highly) significantly between each group. For our analysis, T1 and T2 were grouped, and T3 and T4 were grouped. A *p*-value ≤ 0.05 was considered statistically significant, and a *p*-value ≤ 0.001, highly significant. For the visualization of results, we used Box–Whisker plots. For the statistical analysis, the SPSS23 statistical software package (SPSS Inc., Chicago, IL, USA) was applied.

## 3. Results

### 3.1. Patients’ Cohort

In the analyzed cohort, 15.6% (*n* = 7) of the patients were female, and 84.4% (*n* = 38) were male (Table 1). The age range was from 38 to 71 years, with a mean age of 54.2 (±8.13) years (Table 1). To assess the influence of neoadjuvant radiochemotherapy (RCT) on the staging and histomorphology of OSCC, we analyzed the samples pre and post RCT. The clinical analysis of the pre-RCT cT-status identified approximately 31% cT2 and 42% cT4; post RCT, most samples were pathologically classified as ypT0 (approximately 62%) and ypT1 (22%) (Table 1). Expression analyses in biopsy samples revealed no significant differences between ypT0 and ypT+ cases for CD68, CD163, GLUT1, HIF-1α and TGF-β.

For further analysis, we grouped T1 and T2 and grouped T3 and T4. Comparing the N-status pre and post RCT, we found that around 67% were cN+ and 24% were cN0 pre RCT, whereas the percentage of histologically confirmed pN0 after RCT was around 78% (Table 1). About 70% of the biopsy samples prior to RCT were classified as G2, while only about 22% remained G2 after RCT (Table 1). A histological assessment after RCT led to significant downstaging in OSCC cases compared to the pre-therapeutic clinical staging with a percentages of 76% pre and 9% post RCT being classified in stage 4 (Table 1). More detailed information about the patient’s cohort can be found in Table 1.

### 3.2. CD68

**Figure 2:**
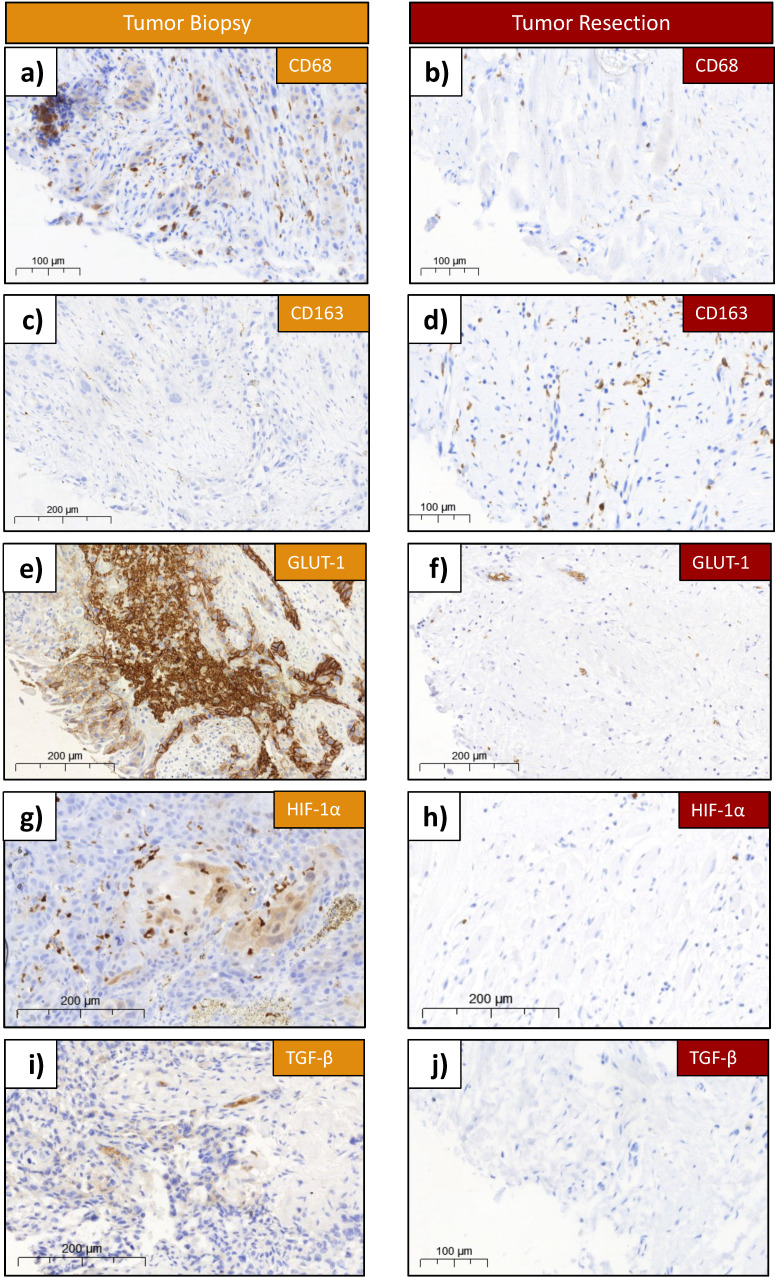
Antibody-stained tissue samples of biopsy and resection. (**a**) Example of CD68 staining in biopsy. (**b**) Example of CD68 staining in resection. (**c**) Example of CD163 staining in biopsy. (**d**) Example of CD163 expression in resection. (**e**) Example of GLUT-1 expression in biopsy. (**f**) Example of GLUT-1 expression in resection. (**g**) Example of HIF-1α expression in biopsy. (**h**) Example of HIF-1α expression in resection. (**i**) Example of TGF-β expression in biopsy. (**j**) Example of TGF-β expression in resection. The figure shows examples of a tissue specimen with the staining of Glut1, HIF-1α, TGF-β, CD68 and CD163. Biopsies are shown on the left, and the resections of the same patient on the right. On each sample, the biomarker and the size scale are labeled. Each slide was scanned with the Pannoramic 250 Flash II scanner (3D Histech, Budapest, Hungary).

For the assessment of the CD68 expression, 27 samples were available in the biopsy group (pre RCT), and 34 in the resection group (post RCT). Exemplary histological slides of the staining before and after therapy are shown (Figure 2a,b). The epithelial compartments of the biopsy is much bigger, compared to the ones in the tumor resection samples post RCT (Figure 2a,b). In contrast, samples post RCT (tumor resection) showed either no epithelial tumor cells at all (ypT0) or small islands of tumor cells in a larger proportion of stroma (Figure 1). A higher density of CD68-expressing cells was demonstrated in the biopsies (Figure 2a).

**Figure 3:**
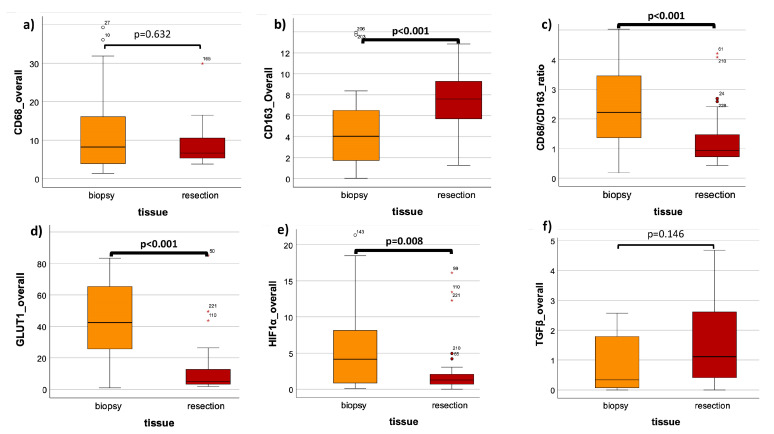
Comparison of tissue’s overall LIs in biopsy (pre RCT) and resection (post RCT). (**a**) CD68 expression in biopsy versus resection. (**b**) CD163 expression in biopsy versus resection. (**c**) CD68/CD163 ratio of expression in biopsy versus resection. (**d**) GLUT-1 expression in biopsy versus resection. (**e**) HIF-1α expression in biopsy versus resection. (**f**) TGF-β expression in biopsy versus resection. The boxplots show the labelling indices (LIs) of CD68 (**a**), CD163 (**b**), the CD68/CD163 ratio (**c**), GLUT-1 (**d**), HIF-1α (**e**) and TGF-β (**f**) in OSCC biopsy (orange) and OSCC resection (red). The analyzed tissue compartment was both the epithelial tumor compartment and the tumor stroma, the overall specimen. The Man–Whitney U test was used for the statistical analysis. The significant *p*-values are marked bold.

**Figure 4:**
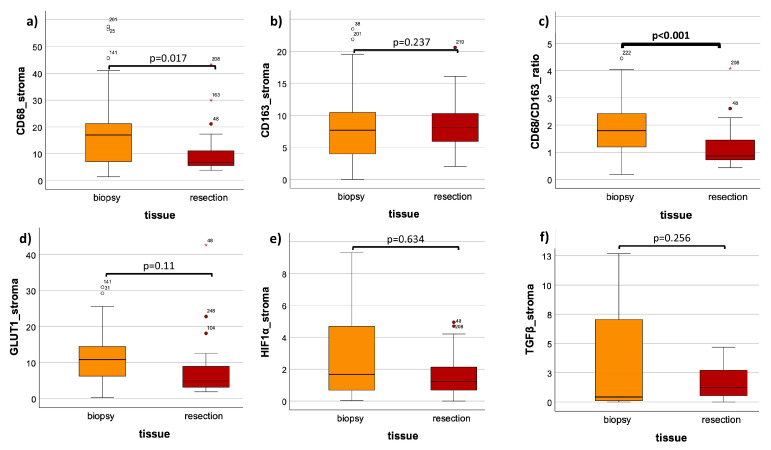
Comparison of tissue stromal LIs in biopsy (pre RCT) and resection (post RCT). (**a**) CD68 expression in biopsy versus resection. (**b**) CD163 expression in biopsy versus resection. (**c**) CD68/CD163 ratio of expression in biopsy versus resection. (**d**) GLUT-1 expression in biopsy versus resection. (**e**) HIF-1α expression in biopsy versus resection. (**f**) TGF-β expression in biopsy versus resection. The boxplots show the labelling indices (LIs) of CD68 (**a**), CD163 (**b**), the CD86/CD163 ratio (**c**), GLUT-1 (**d**), HIF-1α (**e**) and TGF-β (**f**) in OSCC biopsy (orange) and OSCC resection (red). The analyzed tissue compartment was the stroma only. The Man–Whitney U test was used for the statistical analysis. The significant p-values are marked bold.

The mean overall LI in the biopsy was 13.3 (±13.1) and 9.4 (±7.8) in resection, showing no significant expression differences between these two groups (*p* = 0.632) (Figure 3a, Table 2). In analyzing only the stromal compartment, a significant (*p* = 0.017) decrease in the CD68 cell density in tumor resection specimens post RCT (mean LI: 9.9 (±8.1)) was observable compared to the pre-therapeutic biopsies (mean LI: 18.1 (±15.5)) (Figure 4a, Table 2). Comparing the grouped T-status and N-status in biopsy samples, no significant expression differences of CD68 could be detected; detailed values can be found in Table 3.

### 3.3. CD163

For the analysis of the tissue expression of CD163, 34 patients were included. From the biopsy and resection groups, 27 and 34 specimens, respectively, of the same patients were analyzed. Exemplary histological slides of the staining before and after therapy are shown (Figure 2c,d). The epithelial compartment of the biopsy was much bigger, compared to the one in the tumor resection post RCT (Figure 2c,d). A higher density of CD163-expressing cells was demonstrated in the resection (Figure 2c,d). The analysis of the overall LI showed a highly significant (*p* < 0.001) increase in expression after therapy, with a mean LI of 5.2 (±5.2) in biopsy and a mean LI of 8.8 (±6.8) in resection (Figure 3b, Table 2). A Mann–Whitney U test of the stromal part of the biopsy versus resection could not show significant differences (*p* = 0.237) (Figure 4b, Table 2). The mean stromal LI of the biopsy was 8.1 (±5.9), and the mean stromal LI of the resection was 9.5 (±6.8) (Figure 4b, Table 2). Comparing the grouped T-status and N-status pre RCT, no significant expression differences of CD163 could be detected; detailed values can be found in Table 3.

### 3.4. CD68/CD163 Ratio

The ratio of CD68/CD163 was analyzed in both epithelial and stromal parts and exclusively in stroma only (Figure 3c and Figure 4c, Table 2). The overall expression ratio of the biopsy showed a highly significant (*p* < 0.001) decreased CD68/CD163 ratio after therapy (Figure 3c, Table 2). The mean CD68/CD163 ratio pre therapy was 40.8 (±192.6), and the mean CD68/CD163 ratio post therapy was 1.3 (±0.9) (Figure 3c, Table 2). In the stromal compartment, the mean expression ratio of the biopsy was 2.3 (±1.9), and the mean expression ratio of the resection was decreased to 1.2 (±0.8), leading to a highly significant expression ratio change when comparing pre and post RCT (*p* < 0.001) (Figure 4b, Table 2).

### 3.5. GLUT-1

Exemplary histological slides of the GLUT-1 staining before and after therapy are shown (Figure 2e,f). A higher density of GLUT-1-expressing cells was demonstrated in the biopsies (Figure 2e,f). In the expression analysis of 32 patients, including 26 biopsy and 32 resection samples, specimens were again subdivided into overall (stroma + epithelium) and stroma only (Figure 3d and Figure 4d, Table 2). The Mann–Whitney U analysis resulted in a highly significant expression difference in the overall LI between biopsy and resection (*p* < 0.001) (Figure 3d, Table 2). The overall mean LI of the biopsy was 40.8 (±192.6) and decreased to 1.3 (±0.9) after RCT (Figure 3d, Table 2). The analysis of the stromal compartment showed a significant (*p* = 0.011) decrease in the expression in tumor resection specimens post RCT (mean LI: 7.4 (±8.0)) compared to the pre-therapeutic biopsies (mean LI: 11.4 (±8.1)) (Figure 4d, Table 2).

In addition to the comparison of biopsy and resection samples, an analysis of the GLUT-1 LI in biopsy samples, depending on the cT status, was performed (Table 3). The cT1 and cT2 group included 9 patients in total, whereas the cT3/cT4 group consisted of 16 patients (Figure 4, Table 3). Significant expression differences (*p* = 0.020) of Glut1 could be measured within the two groups, with higher protein expressions in T1 and T2 (LI = 60.4 (±20.1) compared to T3 and T4 (LI = 36.3 (±24.6) (Table 3). The analysis of the N-status (N+ and N0) did not indicate any significant expression differences between both groups in stroma, epithelium or overall tissue (Table 3). Detailed information can be found in Table 3.

### 3.6. HIF1α

For the assessment of the HIF1α expression pre RCT (*n* = 25) and post RCT (*n* = 34), 34 patients were included. Exemplary histological slides of the staining before and after therapy are shown (Figure 2g,h). A lower number of HIF1alpha-expressing cells was demonstrated in the resection compared to the harvested sample before therapy (Figure 2g,h). The analysis of the overall and stromal LI showed only significant differences in the overall LI (Figure 3e and Figure 4e, Table 2). The mean overall LI of the biopsy was 6.5 (±7.6), compared to a decreased mean LI of 2.5 (±3.8) after therapy (*p* = 0.008) (Figure 3e, Table 2). The stromal compartment displayed no significant protein expressions of HIF1α between both groups, but a decrease in LI could be measured post RCT (*p* = 0.634) (Figure 4e, Table 2). In stroma, the mean LI of the biopsy was 5.6 (±11.0), compared to a mean LI of 2.6 (±4.0) in the resection samples (Figure 4e, Table 2). Comparing the HIF1α expression of the grouped T-status and N-status before therapy, no significant expression differences could be detected in T1 + T2 versus T3 + T4 or N0 versus N+, neither in stroma nor in epithelium nor in the overall specimen (Table 3). Detailed values and numbers can be found in Table 3.

### 3.7. TGF-β

Exemplary histological slides of the TGF-β staining pre and post therapy are shown (Figure 2i,j). More TGF-β-expressing cells were observed in the biopsy specimens (Figure 2i,j). For the analysis of the expression of TGF-β in tissue specimens of OSCC, 24 specimens in the biopsy group and 34 specimens in the resection group of the same patients were analyzed (Figure 3f and Figure 4f, Table 2). In comparing the mean overall LI, the result was an increased LI of 3.0 (±6.3) in the biopsy compared to a LI of 1.6 (±1.4) post RCT (Figure 3f, Table 2). These values could not show significant expression differences between these two groups (*p* = 0.146) (Figure 3f, Table 2). In comparing only the stromal part of the samples, also no significant expression differences (*p* = 0.256) in the TGF-β expression between both groups could be measured (Figure 4f, Table 2). In stroma, the mean LI of the biopsy samples was 4.8 (±8.8), followed by a mean LI of 1.7 (±1.4) after therapy (Figure 4f, Table 2). In comparing the expression of TGF-β in between the T-status and N-status in the tissue biopsy, no significant expression differences in the TGF-β could be detected in both histomorphological parameters, neither in stroma nor in epithelium nor in the overall tissue (Table 3). Detailed information can be found in Table 3.

## 4. Discussion

In the current study, we were able to show that neoadjuvant RCT with a normofractionated dose of approximately 50 Gy leads to significant immunological and metabolic changes in the tumor microenvironment of OSCC. A major finding was a decrease in CD68-expressing macrophages, which was observed in the whole tumor tissue as well as in the stromal compartment. Statistical significance was reached only in the stromal compartment. In contrast, the cell density of M2-polarized macrophages expressing CD163 increased after neoadjuvant RCT, with a significant difference reached when comparing the entire sample. The CD68/CD163 expression ratio decreased significantly in both compartments in response to neoadjuvant therapy, indicating a shift toward the M2 polarization of macrophages. As those act as tumor-promoting and are associated with tumor progression and metastases [8,9], this expression change can be interpreted as a potential negative effect of neoadjuvant RCT and motivates the evaluation of new dosing and fractionation regimes. This is of special importance if RT or RCT is combined with IT using ICI, as M2 macrophages are potentially associated with inferior responses to ICI therapy [28,29].

A recent study investigated the effect of neoadjuvant immunotherapy in patients with stage III and IV OSCC using the anti-PD1 ICI Nivolumab in combination with 24 Gy of RT (1.8–2 Gy 5× weekly) and chemotherapy with cisplatin and 5-fluorouracil [30]. This retrospective single-arm study—including 30 patients—could show a major pathological response rate of 60% and a disease-free survival of about 70% after 24 months as well as a tolerable toxicity [30]. These results indicate that the addition of low-dose RCT to neoadjuvant ICI IT is a considerable option in the treatment protocol of OSCC. The reported response rate is superior compared to previously published single-agent ICI protocols [30,31]. However, the exact timing of RT is still a matter of debate and needs to be further analyzed in prospective studies.

Another phase 1 study applied hypo-fractionated neoadjuvant RT with up to 3 × 6 Gy in combination with the PD-L1 inhibitor Durvalumab in 21 HPV negative HNSCC patients [32]. A major or complete pathologic response could be achieved in 75% of the cases [32]. The study could show a significant association between therapy response and the immune composition in the tumor microenvironment and the tumor bed. Increased antigen presentation and effector T-cells as well as decreased immunosuppressive cells were associated with therapy response [32].

In another study on HNSCC a neoadjuvant dose of either 40 Gy in five fractions or 24 Gy in three fractions was applied during one week selectively to the primary tumor in combination with the PD1-inhibitor Nivolumab prior to surgical treatment [5]. Nivolumab was neoadjuvantly administrated three times, and surgery was performed six weeks after the initiation of treatment. In the 21 treated patients, a pathologic complete response could be achieved in 67% of cases [5]. This response rate is higher compared to those of neoadjuvant strategies with checkpoint inhibitors alone [5]. However, it needs to be considered that only five patients in this study were HPV negative with only two OSCC cases [5].

Different immune cell types show different sensitivity to radiation doses. In this regard, monocytes were the most radioresistant immune cells [33]. Differences in radiation dose and fractionation result in different immune-modulatory effects [34]. There is evidence that hypo-fractionated protocols are superior with regard to anti-tumoral immune-modulation, especially in combination with ICI immunotherapy [34]. In this context, a sub ablative fractionization regimen of 3 × 8 Gy is often considered to be particularly effective based on preclinical data [2,34]. Overall, there is evidence that lower doses of radiation predominantly act as immune-modulatory while higher doses lead to the increasing cell death of immune cells [34].

Currently, there are several studies analyzing neoadjuvant radioimmunotherapy with different radiation doses and in combination with single- or dual-ICI treatments with or without the addition of chemotherapy in lung cancer [35]. In most of these studies, low-dose therapies and hypofractionation are applied [35]. In this extremely heterogenous study landscape, it is important to gain basic science data on the immunomodulatory effect of RT/RCT to obtain a better scientific rational for defining all parameters to perform clinically successful treatment studies.

In combination therapies, RT should induce a favorable immune microenvironment. This is characterized by an immunogenic cell death, the induction of dendritic cell maturation and T-cell activation [2]. A suboptimal response to radiotherapy is considered to be associated with immunosuppressive cytokines and the M2 polarization of macrophages [2]. As we could detect a shift toward M2 polarized macrophages, it might be an indicator that the conventional fractionization with a total dose of 50 Gy applied in the current study might be suboptimal for achieving immuno-stimulatory effects.

In colorectal cancer, an increase in the tumor mutational burden through neoadjuvant RCT has been shown [26]. This finding is interesting, as an increased tumor mutational burden is generally believed to be associated with an improved response to ICI treatment [36]. An in silico analysis of RNA sequencing data revealed an increased expression of CD8+ T-cells as well as M2 macrophages in advanced rectal cancer in response to neoadjuvant RCT with 25 × 1.8 or 2 Gy in addition to different chemotherapeutic agents [26]. These results are in accordance with the results of the current study showing a shift toward the M2 polarization of macrophages in response to neoadjuvant chemotherapy in OSCC.

There is evidence that it is beneficial to perform an isolated radiotherapy of the primary tumor site without involving the tumor-draining lymph nodes when RT is combined with ICI therapy, and the potential immune stimulating effects of RT should be harnessed [37]. Avoiding the irradiation of the tumor-draining lymph nodes in HNSCC led to an increased rate in lymph node recurrences [2]. However, this effect could be reversed by performing an elective neck dissection without an RT of the tumor-draining lymph nodes [2,38]. It needs to be considered that neck dissection has to be applied after the use of ICI immunotherapy to not reduce the ICI efficiency [2,39]. The potential negative immunologic effects of the RT of the lymphatic drainage are relevant as this was performed in the patient cohort analyzed in the current study.

The contact of macrophages to irradiated cancer cells was shown to polarize them toward M2 [2]. This is problematic as potential combination therapies between ICI and toll-like receptor (TLR) activating agents fail due to an anti-inflammatory response of M2 macrophages toward TLR stimulation [2]. In this regard, the observed shift toward M2 after neoadjuvant RCT can be considered problematic and should be addressed in future neoadjuvant RT and radio-immune therapy protocols.

In addition to changes to macrophage infiltration and polarization, the current study could also show changes to the parameters of glucose metabolism in the microenvironment of OSCC in response to neoadjuvant RCT. A significant decrease in GLUT-1 was observed in all analyzed compartments. In addition, HIF-1α was decreased in response to therapy while statistical significance was reached in the overall analyzed area.

GLUT-1 increases glucose uptake and enables cancer cells to meet their energy demand also in hypoxic conditions leveraging the Warburg effect [22]. In this regard, HIF-1α can synergistically promote radio resistance and induce tumor progression. HIF-1α can increase the invasiveness of cancer cells, promote metastatic spread and radio resistance [22]. In laryngeal cancer, HIF-1α overexpression has been associated with lymph node metastasis, a high T-stage and poor survival [22]. In vitro analyses revealed that the knockout of GLUT-1 and/or HIF-1α reduces tumor growth and radio resistance [22]. This indicates a tumor-biological positive effect of reduced GLUT-1 and HIF-1α expressions.

In addition to cancer cells, HIF-1α is also expressed in macrophages and is involved in macrophage activation [23]. In macrophages, the HIF-1α expression is associated with M1 polarization [23]. In T-cells, HIF-1α shows the highest expression in pro-inflammatory Th17 cells, while immunosuppressive T_reg_ cells show the lowest HIF-1α levels [23]. The decrease in HIF-1α observed in the current study could also be associated with the decrease in predominantly M1-polarized CD68 positive macrophages and the shift toward CD163-associated M2 polarization.

In OSCC tissue, a correlation between a high GLUT-1 expression and inferior prognosis has been reported [25,40]. In a mouse model, RT led to a decreased GLUT-1 expression in OSCC tumor tissue [25]. A clinical analysis in a neoadjuvant-treated patient cohort by Mohr et al. revealed that an increased GLUT-1 expression was associated with increased resistance toward neoadjuvant RCT [41]. Similar results were seen in a Japanese OSCC patient cohort treated with neoadjuvant RCT [42]. The data of our current analysis show that neoadjuvant RCT itself can reduce the GLUT-1 expression; the effect was specifically observed in the overall analyzed samples as well as in the peritumoral stroma. These data indicate that neoadjuvant RCT also reduces the GLUT-1 expression and eventually also glucose metabolism in the tumor stroma. Interestingly, smaller T1/T2 tumors showed significantly higher GLUT-1 expressions compared to T3/T3 OSCC. This might indicate a potentially inferior response to RT or RCT in this group.

The current study could not show significant changes in the TGF-β expression in response to neoadjuvant RCT. The role of TGF-β signaling is complex, as on the one hand, it can act as tumor-promoting, and on the other hand, the cytokine also has anti-tumoral actions [18]. In early OSCC carcinogenesis, TGF-β is believed to have anti-proliferative effects. In later stages of carcinogenesis and established tumors, the immunosuppressive effects of TGF-β are believed to be dominant and act as tumor-promoting [18]. In OSCC cultures, tumor cell-derived TGF-β has been shown to drive macrophages toward M2, and TGF-β blocking could reverse this effect [43]. In previous work, our group showed that TGF-β is induced in irradiated blood vessels [44], which contributes to the well-known phenomenon of radiation-associated fibrosis [45]. There are ongoing studies investigating dual ICI therapy in HNSCC and OSCC with anti-PD-L1 and TGF-β targeting, which could lead to better responses [46]. In this regard, further studies are necessary to assess the effect of RT, especially in combination with combined PD1/PD-L1 and TGF-β targeting.

### Limitations of the Study

This retrospective study has some limitations. There was heterogeneity among the patients, as there were 7 female and 38 male patients. This phenomenon can be explained by the gender-dependent distribution of OSCC. Thus, 75% of new cases in Germany are men, which is similar to the distribution in our cohort [6].

In addition, all patients analyzed in the current study were treated between the years 1997 and 2003. This could influence the reliability of the documented clinical parameters of the study collective.

An analysis of all biomarkers was possible in the overall available specimen area as well as in the tumor stroma compartment. An isolated assessment of the epithelial tumor compartment was possible only in the pre-therapeutic biopsies. In the tumor resection specimens, in many cases, there was no vital tumor available (ypT0). In cases with available residual tumor after neoadjuvant RCT, there were only very small areas of epithelial tumor cell available. Therefore, it was decided not to analyze the epithelial compartment separately in the tumor resection specimens. In addition, biopsy or tumor resection samples were not available or not analyzable in some cases.

A further limitation is the fact that all observed changes cannot be attributed to RT or to the concurrent chemotherapy.

## 5. Conclusions

Neoadjuvant radiochemotherapy with approximately 50 Gy in a normofractionated manner leads to significant changes in the immunologic and metabolic microenvironment of OSCC. The relatively high-dose of radiation applied in the patient cohort analyzed in the current study is associated with potentially immunologic negative effects involving a shift of macrophage polarization toward tumor-promoting M2 cells. This needs to be reflected, especially if new concepts on the neoadjuvant radiotherapy of OSCC are considered, which are mostly applied in alternative fractionization schemes, lower doses and in combination with immune checkpoint inhibitors. Further basic science studies are needed to better understand the immuno-oncological effects of all these parameters in order to find the optimal multimodal neoadjuvant treatment regimen for OSCC.

## Figures and Tables

**Table 1:** Demographics of the cohort. The table shows the characteristics of the 45 patients in our study cohort. The parameters are sex, age, T-status, N-status, grading and staging. Prior to radio chemotherapy (pre RCT), the cT- and cN-statuses were indicated, as well as the pT- and pN-statuses after RCT (post RCT). The included cases are given in percentage (%) and in total number (*n*). The percentages are rounded and therefore do not add up to 100%.

		Total Number of Cases: 45			
Cases		*n*	% of Cases		
Sex	Female	7	16		
	Male	38	84		
Mean age		54.22 (SD 8.13)			
Age range		38–71 years			
		Pre RCT (Biopsy)		Post RCT (Resection)	
		*n*	%	*n*	%
T-status	T0	0	0	28	62
	T1	2	4	10	22
	T2	14	31	0	0
	T3	6	13	1	2
	T4	19	42	5	11
	unknown	4	9	1	2
N-Status	N0	11	24	35	78
	N+	30	67	9	20
	unknown	4	9	1	2
Grading	G1	5	11	1	2
	G2	31	69	10	22
	G3	7	16	6	13
	G4	1	2	1	2
	unknown	1	2	27	60
Staging	1	1	2	8	18
	2	5	11	0	0
	3	4	9	3	7
	4	34	76	4	9
	unknown	1	2.2	30	67

**Table 2:** Labeling indices in biopsy versus resection. The table shows the labeling indices (LIs) for GLUT-1, HIF-1α, TGF-β, CD68 and CD163 in oral squamous cell carcinoma (OSCC) pre RCT in biopsy specimens compared to post RCT in tumor resection specimens. LIs in the tumor stroma, and the overall tissue specimen (epithelial + stroma), were analyzed. In addition, the CD68/CD163 expression ratio is provided. A Man–Whitney U test was used for the statistical analysis. *p*-values are marked in bold letters.

Marker	Tissue	Compartment	*n*	Mean	Median	SD	*p*-Value
CD68	biopsy	overall	27	13.3	08.2	13.1	0.632
	resection		34	09.4	06.7	07.8	
	biopsy	stroma	27	18.1	17.0	15.5	**0.017**
	resection		34	09.9	06.7	08.1	
CD163	biopsy	overall	27	05.2	04.0	05.2	**<0.001**
	resection		33	08.8	07.6	06.8	
	biopsy	stroma	27	08.1	07.7	05.9	0.237
	resection		33	09.5	08.1	06.8	
CD68/CD163	biopsy	overall	27	40.1	02.2	192.6	**<0.001**
	resection		33	01.3	00.9	00.9	
	biopsy	stroma	26	02.3	01.8	01.9	**<0.001**
	resection		33	01.2	00.9	00.8	
GLUT-1	biopsy	overall	26	44.4	42.4	25.2	**<0.001**
	resection		32	12.4	05.1	17.4	
	biopsy	stroma	26	11.4	10.8	08.1	**0.011**
	resection		32	07.4	04.9	08.0	
HIF-1α	biopsy	overall	25	06.5	04.2	07.6	**0.008**
	resection		34	02.5	01.3	03.8	
	biopsy	stroma	25	05.6	01.7	11.0	0.634
	resection		34	02.6	01.2	04.0	
TGF-β	biopsy	overall	24	03.0	00.3	06.3	0.146
	resection		34	01.6	01.1	01.4	
	biopsy	stroma	24	04.8	00.4	08.8	0.256
	resection		34	01.7	01.2	01.4	

**Table 3:** Labeling indices in biopsy samples. The table shows the labeling indices (LIs) for GLUT-1, HIF-1α, TGF-β, CD68 and CD163 in oral squamous cell carcinoma (OSCC) biopsies. The expressions of the aforementioned markers are compared between the low and advanced T-status (T1/T2 vs. T3/T4) and N-status (N0 vs. N+) in the epithelial tumor compartment, the tumor stroma and the overall tissue specimen (epithelial + stroma). A Man–Whitney U test was used for the statistical analysis. *p*-values are marked in bold letters.

Biopsy		CD163				CD68				GLUT-1				HIF-1α				TGF-β			
		*n*	Mean	SD	*p*-Value	*n*	Mean	SD	*p*-Value	*n*	Mean	SD	*p*-Value	*n*	Mean	SD	*p*-Value	*n*	Mean	SD	*p*-Value
T-status	Epithelium	26			0.833	26			0.287	25			0.121	24			0.928	23			0.548
	T1-T2	9	1.3	1.8		9	7.8	11.0		9	75.7	17.4		8	6.2	5.1		8	2.6	5.0	
	T3-T4	17	3.0	6.2		17	12.6	13.5		16	58.4	27.3		16	6.3	5.9		15	4.1	7.5	
	Stroma	26			0.958	26			0.751	25			0.095	24			0.881	23			1.00
	T1-T2	9	7.3	3.8		9	17.6	16.5		9	60.4	20.1		8	4.1	5.8		8	3.9	7.3	
	T3-T4	17	8.8	6.9		17	18.3	15.9		16	36.3	24.6		16	6.6	13.2		15	5.7	9.8	
	Overall	26			0.287	26			0.458	25			**0.020**	24			0.787	23			0.548
	T1-T2	9	3.6	3.0		9	10.8	11.9		9	60.4	20.1		8	6.1	6.2		8	1.7	3.2	
	T3-T4	17	6.2	6.0		17	14.6	14.2		16	36.3	24.6		16	6.3	8.5		15	3.8	7.5	
N-status	Epithelium	25			0.642	25			0.475	24			0.415	23			0.812	22			0.218
	N0	9	2.8	5.9		19	11.2	13.0	N0	18	65.5	22.8		17	6.6	5.6		17	3.9	7.1	
	N+	16	1.3	1.9		6	6.8	9.2		6	68.7	33.0		6	7.5	6.8		5	3.0	6.3	
	Stroma	25			0.514	25			1.000	24			0.310	23			0.919	22			0.140
	N0	9	8.9	6.4		19	18.7	17.0		18	11.4	8.4		17	4.4	7.0		17	5.6	9.3	
	N+	16	6.4	4.7		6	17.8	14.6		6	13.5	8.1		6	10.4	19.5		5	4.2	9.1	
	Overall	25			0.366	25			0.828	24			0.251	23			0.812	22			0.189
	N0	9	5.8	5.8		19	13.7	13.9		18	43.2	22.9		17	6.1	6.2		17	3.6	7.1	
	N+	16	3.3	3.2		6	11.8	12.5		6	55.8	30.2		6	9.1	11.9		5	2.0	4.1	

## Data Availability

The primary data of this study are available from the corresponding author upon request.
